# Bridge Displacement Estimation Using a Co-Located Acceleration and Strain

**DOI:** 10.3390/s20041109

**Published:** 2020-02-18

**Authors:** Muhammad Zohaib Sarwar, Jong-Woong Park

**Affiliations:** 1 Department of Structural Engineering, Norwegian University of Science and Technology, NO-7491 Trondheim, Norway; muhammad.z.sarwar@ntnu.no; 2 School of Civil and Environmental Engineering, Chung-Ang University, Dongjak, Seoul 06974, Korea

**Keywords:** structural health monitoring, sensor fusion, adaptive Kalman filter, displacement estimation, reference-free displacement

## Abstract

Structural displacement is an important metric for assessing structural conditions because it has a direct relationship with the structural stiffness. Many bridge displacement measurement techniques have been developed, but most methods require fixed reference points in the vicinity of the target structure that limits the field implementations. A promising alternative is to use reference-free measurement techniques that indirectly estimate the displacement by using measurements such as acceleration and strain. This paper proposes novel reference-free bridge displacement estimation by the fusion of single acceleration with pseudo-static displacement derived from co-located strain measurements. First, we propose a conversion of the strain at the center of a beam into displacement based on the geometric relationship between strain and deflection curves with reference-free calibration. Second, an adaptive Kalman filter is proposed to fuse the displacement generated by strain with acceleration by recursively estimating the noise covariance of displacement from strain measurements which is vulnerable to measurement condition. Both numerical and experimental validations are presented to demonstrate the efficiency and robustness of the proposed approach.

## 1. Introduction

Structural displacement is one of the most important metrics for evaluating the serviceability and integrity of bridge structures because it is directly related to loadbearing capacity [1,2,3]. However, measuring the displacement of bridge structures is a challenging task because of a lack of appropriate sensors. Conventionally, displacement measurement is carried out using a linear voltage differential transformer (LVDT) [4,5] or linear doppler velocimeter (LDV). An LVDT is a contact sensor that directly measures the displacement and provides high precision, but practical implementation on large-scale structures is limited because one end of an LVDT must be attached to a fixed reference while the other end is attached to a target structure. Therefore, LVDTs cannot be employed for long-term structural health monitoring [6]. An LDV is a non-contact sensor with high precision, but it also requires a fixed reference because it measures relative displacement from the sensor to the structure, which hinders long-term measurement. Additionally, the high cost of LDVs is a limiting factor for widespread adoption for ambient displacement monitoring. 

As an alternative to traditional measurement methods, acceleration measurements can be used for reference-free measurement systems. The reference-free displacement estimation using acceleration can be performed through direct integration in the time or frequency domains [3,7,8,9]. For practical applications, researchers have designed finite impulse response (FIR) filters for the numerical integration of acceleration in the frequency domain while filtering low-frequency components below the first dominant frequency of a target structure [10]. FIR filters are computationally inexpensive and easy to implement in real-world applications. However, only dynamic displacement can be reconstructed from FIR-filter-based methods to avoid the drift resulting from numerical integration [11,12,13,14]. Hybrid reference free total displacement system is also proposed but methods are limited because of requirement of finite element model of the structure. [15,16]. 

In addition to acceleration, reference-free displacement can also be reconstructed from a set of strain measurements based on strain-displacement relationships, such as modal transformations and geometric relationships [17,18,19,20]. Strain-based displacement methods reconstruct pseudo-static displacement very well, but the most critical part of transformation is to obtain the unknown location of a neutral axis that determines the magnitude of the resulting displacement.

To measure the complete displacement with both pseudo-static and dynamic components, a Kalman filter, which is one of the most widely used filters for sensor fusion, can be adopted. Kalman filters have been employed to obtain optical estimates of displacement based on the numerical integration of acceleration and pseudo-static displacement acquired from global positioning systems [21] or LDVs [22,23]. The Kalman filter has been successfully implemented to displacement fusion between acceleration and direct displacement where measurement noise (R) and process noise (Q) can be clearly identified [9]. However, in the case of reference-free displacement estimation where R and Q may not be determinate, the performance of a Kalman filter is not guaranteed [24,25,26]. 

In this paper, structural displacement estimation based on the fusion of acceleration and strain is proposed. First, strain-based displacement estimation method, and reference-free calibration that calibrates the scale of strain-based displacement by minimizing the second differential of strain-based displacement and measured acceleration are proposed. In the second stage, acceleration and strain-based displacement are integrated using an adaptive Kalman filter to recursively fuse acceleration with strain-based displacement while updating noise covariance of strain-based displacement data which is highly affected by environmental condition [27]. The proposed method is numerically and experimentally validated on simply supported beam structures.

The remainder of this paper is organized as follows. Section 2 describes the proposed approach for displacement measurement, including strain-displacement relationships, reference-free calibration, formulation of a state-space model, and the AKF. Section 3 presents numerical validations of the proposed method on a simply supported beam structure excited by a moving load. Experimental validation on a simply supported prestressed concrete bridge with moving vehicle loading is discussed in Section 4. Finally, Section 5 summarizes our conclusions based on the numerical and experimental results.

## 2. Proposed Approach

### 2.1. Overview

**Figure 1:**
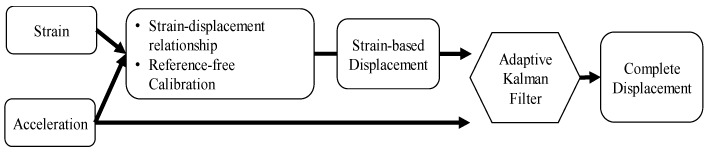
Overview of the proposed method.

The proposed reference-free vertical displacement measurement method using strain and acceleration consists of two phases. First, strain-based displacement is reconstructed from the strain measured at the center of a beam based on the strain-displacement geometric relationships and reference-free calibration, which calibrates the strain-based displacement by referencing the co-located acceleration by minimizing the second-order differential of strain and acceleration measurements. In the second phase, acceleration and strain-based displacement are fused using an AKF (see Figure 1).

### 2.2. Strain-Displacement Relationship for a Simple Beam

For a simple beam with a length of *L*, the derivative equation for the curvature-displacement relationship at a location *x* on the beam is written as [28]

(1) $$\kappa (x)=\frac{d^{2}u(x)}{dx^{2}}$$

where u(x) and κ(x) are the displacement and curvature at *x*, respectively, ranging from zero to *L*. Similarly, the relationship between curvature and longitudinal strain from Euler–Bernoulli beam theory is written as

(2) $$\kappa (x)=-\frac{\varepsilon_{x}}{h_{c}}$$

where $\varepsilon_{x}$ is the strain and $h_{c}$ is the distance from the neutral axis. Based on Equations (1) and (2), the relationship between displacement and strain can be written as

(3) $$\frac{d^{2}u(x)}{dx^{2}}=-\frac{\varepsilon_{x}}{h_{c}}$$

Using the finite difference method, the second-order differential of the displacement in Equation (3) can be expressed as a finite difference equation as [28].

(4) $$\left(\frac{d^{2}u(x)}{dx^{2}}\right)\approx \frac{u_{i+1}-2u_{i}+u_{i-1}}{\Delta x^{2}}=-\frac{\varepsilon_{x}}{h_{c}}$$

where i=[1,2,3,…,N] is the *i-th* discrete location on a beam and Δx is the interval between discrete locations. For a simply supported beam with strain measured at the center of the beam, the displacement can be obtained as

(5) $$u_{s}=\frac{\varepsilon }{2h_{c}}{\left(\frac{L}{2}\right)}^{2}$$

with zero-displacement boundary conditions at the supports. However, for an unknown distance from the neutral axis, the estimated strain-based displacement must be calibrated by a factor of α. Therefore, the calibrated strain-based displacement is expressed as

(6) $$d_{s}=\alpha u_{s}$$

where α is the calibration factor that can be obtained through calibration experiments using a reference displacement sensor. This study used acceleration to calibrate strain-based displacement directly without any additional experiments based on reference-free calibration, which will be discussed in the following section. 

### 2.3. Referecnce Free Callibration

**Figure 2:**
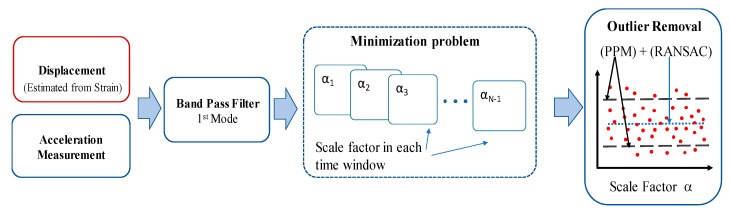
Overview of calibration factor estimation.

For field implementation, calibration using a reference is a required process in existing strain-based approaches [18]. To estimate the calibration factor α discussed above, reference-free calibration is formulated in the time domain based on the equivalent neutral axis estimation method proposed by [29] (see Figure 2).

Reference-free calibration first applies a band pass filter to the acceleration and strain-based displacement measured at the mid-span point to extract the first-mode motions of acceleration and displacement. Next, the relationship between the second derivative of strain-based displacement from Equation (6) and the acceleration at the mid-span point is tuned to satisfy Equation (7).

(7) $$a_{s}=\frac{d^{2}}{dt^{2}}\alpha u_{s}$$

where $a_{s}$ is the second derivative of strain-based displacement measured at the mid-span point and $a_{m}$ is the measured acceleration. The calibration factor *α* can be calculated by minimizing the difference between $a_{s}$ and $a_{m}$ for a time window from *T*_1_ to *T*_2_ as

(8) $$\underset{\alpha }{\min}\Pi_{E}=\frac{1}{2}\int_{T_{1}}^{T_{2}}{\left(a_{s}-a_{m}\right)}^{2}dt$$

For discrete integration, Equation (8) can be approximated using the trapezoidal rule 4

(9) $$\Pi_{E}=\frac{1}{2}{\left\|L_{a}(a_{s}-a_{m})\right\|}_{2}^{2}\Delta t$$

where Δt is the time step and $L_{a}$ is a diagonal matrix with ones for all diagonal entries except the first and last entries, which are equal to $1/\sqrt{2}$. Equation (7) can also be discretized using the central finite difference as

(10) $$a_{s}=\frac{\alpha }{\Delta t^{2}}L_{c}u_{s}$$

where *L_c_* is a second-order derivative operator of order (*N_s_* − 2) × (*N_s_*) [11] and Ns is the number of data points in the time interval from *T*_1_ to *T*_2_. Substituting Equation (10) into Equation (11) yields the following minimization problem in the discrete time domain.

(11) $$\underset{\alpha }{\min}\Pi_{E}=\frac{1}{2}{\left\|\alpha L_{a}L_{c}u_{s}-\Delta t^{2}L_{a}a_{m}\right\|}_{2}^{2}$$

The minimization problem expressed in Equation (11) yields a unique solution for *α*.

(12) $$\alpha ={\left(u_{s}^{T}L^{T}Lu_{s}\right)}^{-1}\left(u_{s}^{T}L^{T}L_{a}\Delta t^{2}\right)a_{m}$$

where $L=L_{a}L_{c}$. Given the strain-based displacement and acceleration, a series of calibration factors can be obtained for each time window defined from *T*_1_ to *T*_2_, whose sizes can be determined based on the first natural frequency of the structure as

(13) $$N_{s}=f_{s}\frac{F_{w}}{f_{T}}$$

where *N_s_* is the number of data in the time window from *T*_1_ to *T*_2_, and *f_T_*, *F_w_*, and *f_s_* correspond to the natural frequency, windowing factor, and sampling rate for measurement, respectively. Note that a windowing factor *F_w_* of three can be used as a standard windowing factor.

The series of calibration factors obtained for each window include outliers generated by measurement noise. To remove the outliers and obtain accurate values of *α*, the calibration factor estimated in each time-window is filtered with peak power matching (PPM) [8] and the random sample consensus (RANSAC) method is applied to find the best estimate of *α* within a predefined number iterations [29].

### 2.4. State Space Formulation for Displacment Fusion

Given the values for acceleration and strain-based displacement, a continuous state-space model for displacement fusion can be defined as

(14) $$\left[\begin{array}{c} \dot{x}\\ \ddot{x} \end{array}\right]=\left[\begin{array}{cc} 0 & 1\\ 0 & 0 \end{array}\right]\left[\begin{array}{c} x\\ \dot{x} \end{array}\right]+\left[\begin{array}{c} 0\\ 1 \end{array}\right]a_{m}+\left[\begin{array}{c} 0\\ 1 \end{array}\right]\eta_{am}$$

(15) $$d_{s}=\alpha u_{s}=\left[\begin{array}{cc} 1 & 0 \end{array}\right]\left[\begin{array}{c} x\\ \dot{x} \end{array}\right]+\eta_{sm}$$

where $a_{m}$ is the measured acceleration and $d_{s}$ is the measured displacement. $\eta_{sm}$ and $\eta_{am}$ are the measurement noises associated with strain-based displacement and acceleration, respectively, and are modeled as Gaussian distributions with covariance values of *r* and *q*, respectively.

To construct the state-space model, displacement and velocity are defined as state variables and a state vector is expressed as

(16) $$x={\left[\begin{array}{cc} x & \dot{x} \end{array}\right]}^{T}$$

The system in Equation (14) and observation in Equation (15) can be expressed as

(17) $$\dot{x}=Ax+Ba_{m}+w$$

(18) $$d_{s}=Cx+v$$

where $w∼(0,Q),\;Q=\left[\begin{array}{cc} 0 & 0\\ 0 & q \end{array}\right]$ and v∼(0,R)=r. The discrete forms of the state-space representations in Equations (17) and (18) are expressed in Equations (19) and (20), respectively, based on the measured acceleration $a_{m}$ and displacement *d_s_*(*k*) in a discrete time domain

(19) $$x(k)=Ax(k-1)+Ba_{m}(k)+w(k-1)$$

(20) $$d_{s}(k)=Cx(k)+v(k)$$

where **A**, **B**, and **C** are defined as

(21) $$A=\left[\begin{array}{cc} 1 & \Delta t\\ 0 & 1 \end{array}\right],\;B=\left[\begin{array}{c} \Delta t^{2}/2\\ \Delta t \end{array}\right],\;C=\left[\begin{array}{cc} 1 & 0 \end{array}\right]$$

w(k) and v(k) are the discrete measurement noise of acceleration and displacement, respectively. The discrete noise covariance matrices for **Q** and **R** are expressed as

(22) $$Q=\left[\begin{array}{cc} \Delta t^{3}/3 & \Delta t^{2}/2\\ \Delta t^{2}/2 & \Delta t \end{array}\right]q,\;R=r[\Delta t^{-1}]$$

where Δt is the sampling time for both acceleration and displacement. 

To fuse acceleration and strain-displacement to estimate real displacement, a straightforward solution is to apply a conventional Kalman filter (CKF). However, the stability and robustness of state estimation heavily depend on the noise covariance values of acceleration *q* and strain-displacement *r*. Although *q* can be obtained from sensor testing or datasheets, *r* cannot be determined because strain is strongly affected by electrical noise. 

To address this issue, an AKF was formulated to estimate the measurement noise covariance recursively at each time step while calculating the best estimate for each state. The formulation of the AKF is presented in the following section. 

### 2.5. Adaptive Kalman filter (AKF)

The formulation of the AKF consists of three steps: initialization, prediction, and correction. In the AKF algorithm, the state vector ${\hat{x}}_{k|k}\in {\mathbb{R}}^{n}$, error covariance $P_{k|k}\in {\mathbb{R}}^{n\times n}$, and residual $s_{k|k}\in {\mathbb{R}}^{m}$ are recursively updated at each time step *k*. For initialization at the initial time step of *k* = 0, the initial state is assumed to be a Gaussian vector defined as $x(0)∼N(x_{0},P_{0})$. Table 1 summarizes the implementation flow for the AKF.

To update the measurement noise covariance **R** in the AKF recursively, we adopted the residual base method proposed by [30]. The residual is defined as the difference between the actual measurement $d_{s(k)}$ and estimated measurement ${\hat{x}}_{k}$ at time step *k*. A residual $s_{k}$ can be expressed as

(23) $$s_{k}=\{d_{s(k)}-C{\hat{x}}_{k}\}$$

Based on a residual *s_k_*, *R_k_* is the discrete form of **R** updated at each time step and is expressed as

(24) $$R_{k}=E\left[s_{k}s_{k}^{T}\right]+CP_{k}^{-}C^{T}$$

Akhlaghi et al. [30] proposed a simplified method for calculating $R_{k}$ by introducing a forgetting factor β, as shown in Equation (25).

(25) $$R_{k}=\beta R_{k-1}+(1-\beta )(s_{k}s_{k}^{T}+CP_{k}^{-}C^{T})$$

where 0<β≤1. The fluctuation in adaptively estimated $R_{k}$ values depends on the value of β. The smaller the value of β, the less weight is given to the previous estimated value, allowing the filter to synchronize with changes after a short delay. In this study, we set β = 0.3 for all analyses.

## 3. Numerical Validation

### 3.1. Numerical Setup

**Figure 3:**
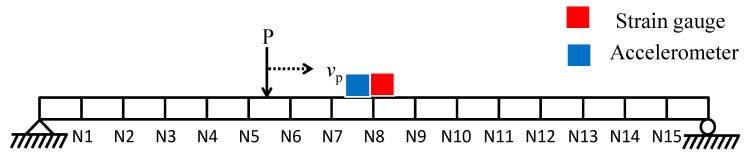
Beam model with a moving load.

Numerical analysis was carried out by exciting the beam by a vertical moving load P with a velocity *v_p_* of 0.2 m/s, as shown in Figure 3. The material properties and dimensions of the beam are summarized in Table 2. The moving load P consists of a static load of 8 N and a zero-mean gaussian random component with a standard deviation of 3 N. The boundary conditions of beam is fixed. Simulations were conducted using MATLAB Simulink to generate strain and acceleration responses in the time domain at N8 (i.e., mid-span point of the beam model). Additionally, 10% root-mean-squared (RMS) noise was added to the acceleration, and four cases of 5%, 10%, 15%, and 20% RMS noise were considered for strain responses.

### 3.2. Results and Discussion

**Figure 4:**
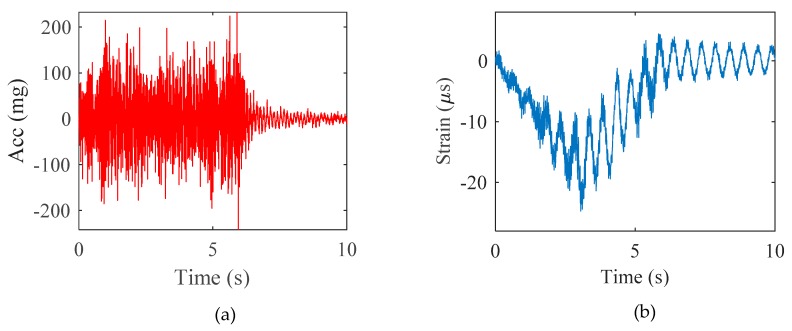
Numerical validation: (**a**) acceleration response and (**b**) strain response.

Figure 4 presents the acceleration and strain measured at the mid-span point. The strain was converted into displacement using geometric relationship in Equation (7) combined with reference-free calibration. The calibration factor α was calculated to be 0.334 m for scaling the strain-based displacement. For calibration, the size of the time window *N_s_* was set based on a standard windowing factor *F_w_* of three and a target frequency for the first natural mode of the structure of 1.986 Hz. To eliminate outliers and identify the best values of α, the calibration factors were first filtered with an upper and lower limit of 0.8 and 1.2 times the PPM value [29], respectively, and RANSAC was performed with the data points and threshold for residuals set to *N_s_*/6 and 0.001, respectively. A total of 10,000 iterations were calculated.

**Figure 5:**
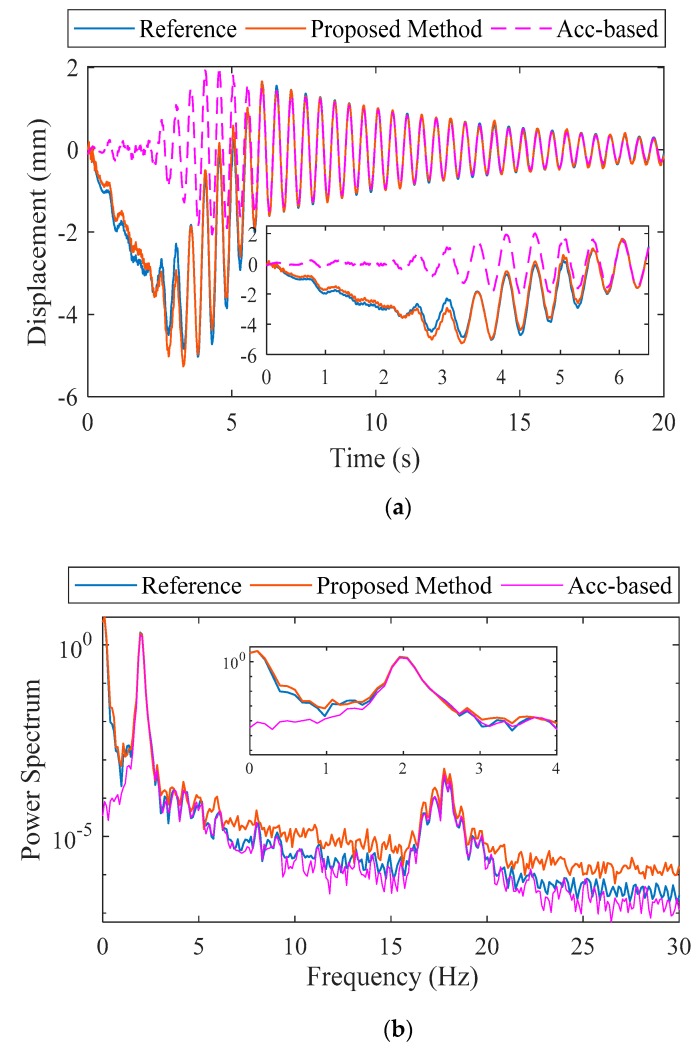
Numerical validation: (**a**) estimated displacement and (**b**) frequency response.

An AKF was applied to fuse the acceleration with strain-based displacement data. The displacement calculated by the proposed method, acceleration-based displacement, and reference displacement are compared in Figure 5 in both the time and frequency domains. It should be noted that the vertical displacement at N8 was extracted numerically for the reference displacement and the acceleration-based displacement was obtained using the FIR filter proposed by Lee et al. [11].

The displacement calculated by the proposed method and the reference displacement show good agreement in both the time and frequency domains. In time domain, the peak displacements from the proposed method and reference displacement were −5.18 mm and −4.929 mm, respectively, representing a discrepancy of only 4.84%. The acceleration-based displacement was only −2.06 mm based on the lack of a pseudo-static component in the displacement. In the frequency domain, the three dynamic displacements show good agreement with peaks at two natural frequencies. Pseudo-static displacement is clearly reconstructed in the results of the proposed method as shown in the zoom view of the Figure 5a,b.

To evaluate the estimation accuracy quantitatively, we utilized the error index defined in Equation (28).

(28) $$E_{rr}=E\left|\frac{\max(u_{est})-\max(u_{ref})}{\max(u_{ref})}\right|$$

where *u_ref_* and *u_est_* are the reference and estimated displacements, respectively, and E[.] is the mean value from 50 simulations of each noise scenario. Note that 50 simulations were conducted to account for the effects of randomness in the measurements and excitations. Table 3 summarizes the accuracy of the proposed method under all four noise scenarios. The proposed method successfully estimated the vertical displacement with an error of 8% under the extreme condition of 20% RMS noise. The results in Table 3 indicate that the proposed method can generally estimate vertical displacement with an error of 5% and can be applied to strain data with an extreme noise level of 20% without adjusting the measurement noise covariance of R.

### 3.3. Validation of Robustness of Proposed Method

To evaluate the robustness of the proposed method, numerical simulations were carried out to investigate the effects $R_{k}$ on the accuracy of displacement estimation. A conventional Kalman filter (CKF) that does not update R and the proposed method were compared. 

**Figure 6:**
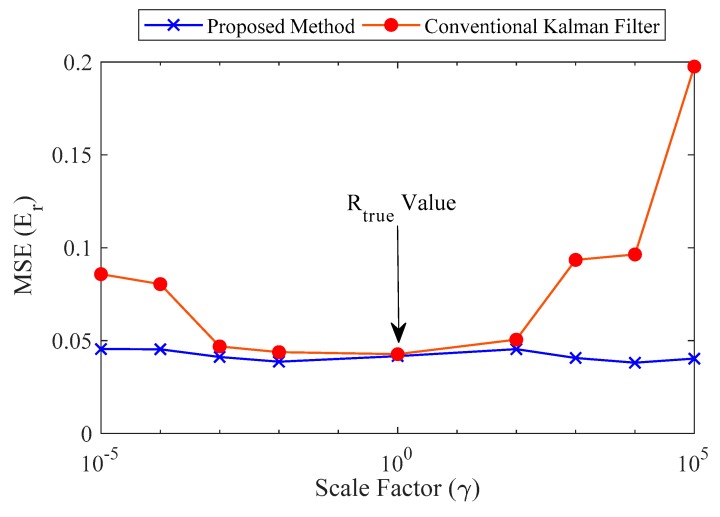
Comparison between the proposed method and a conventional Kalman filter (CKF).

The measurement noise at time $t_{k}$, $R_{k}$ was set by scaling *R_true_* (i.e., true value of measurement noise covariance) by a factor of γ ranging from 10^−5^ to 10^5^. Note that process noise covariance Q can be computed using noise covariance of the acceleration. A total of 50 simulations were conducted with RMS noise of 10% for both acceleration and strain. The robustness of the proposed method was analyzed quantitatively using the error defined in Equation (29).

(29) $$E_{r}=E\left|\frac{\sigma (u_{est}-u_{ref})}{\sigma (u_{ref})}\right|$$

where σ[.] is the standard deviation. Figure 6 reveals the stability of the proposed method compared to a CKF in terms of the noise covariance factor. The accuracy of displacement estimation is strongly affected by the choice of R for the CKF, but the displacement estimates generated by the proposed method show consistent error values over a wide range of γ values. 

Note that when γ is equal to one, the errors of the proposed method and CKF are identical. The findings of our numerical validation can be summarized as follows:

- The proposed method can estimate the vertical displacement of a simply supported beam using a single strain and acceleration measurement at the mid-span point.
- The proposed method reliably estimates vertical displacement, regardless of the noise in strain measurement, by using adaptive filtering

## 4. Experimental Validation

### 4.1. Expereimental Setup

**Figure 7:**
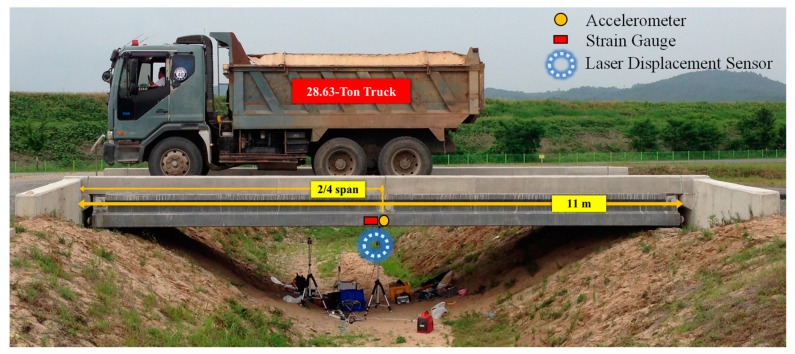
Experimental setup for field validation.

For the validation of field applicability, the proposed method was tested on a pre-stressed concrete bridge located at the test site of the Korean Institute of Construction Technology on the Andong River. A 28.63-ton truck was used for testing two experimental loading cases at speeds of 5 km/h and 15 km/h (see Figure 7).

Sensors for measuring the bridge response were installed at the locations indicated in Figure 7. The strain gauge is installed at the mid-span point under the bridge and the accelerometer is installed in the same location. Note that the location of the mid-span point is 5.63 m from the left end of the bridge. To collect reference signals, a laser displacement sensor is also installed at the mid-span point of the bridge. The complete configuration is presented in Figure 7.

### 4.2. Results

**Figure 8:**
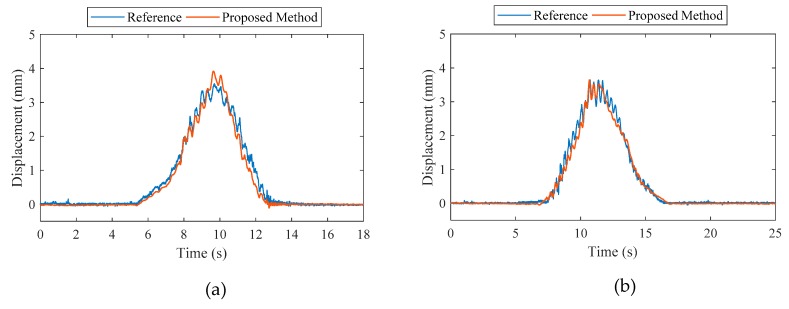
Comparison of maximum displacements at the mid-span point of the bridge: (**a**) truck speed of 5 km/h (**b**) truck speed of 15 km/h.

The proposed method was used to estimate the displacement at the mid-span point of the bridge. The calibration factor for the strain displacement relationship was computed to be 0.0373 m using the relationship discussed in Section 2.2. The forgetting factor for the AKF was set to 0.4 and the measurement covariance noise was initialized to a value of one. The estimated results were compared to the reference values measured by the laser displacement sensor for two different loading cases, as shown in Figure 8.

The reference displacement values, and the values calculated by the proposed method agree well in terms of overall trends in the time domain. When comparing maximum peak displacement values, the reference peak is 3.568 mm and peak calculated by the proposed method is 3.786 mm for the loading case at a speed of 5 km/h. For quantitative estimation, error was computed using Equation (29) for both the loading cases. Table 4 summarizes the errors and maximum deflections calculated by the proposed method relative to the reference. 

From Table 4, the errors computed for the two-loading case are 6.1% and 1.73%, demonstrating that the proposed estimation approach successfully estimate structural displacement using only a single acceleration and strain measurement.

## 5. Conclusions

This paper proposes a bridge displacement measurement method using a co-located acceleration and strain. The proposed method obtains strain-based displacement using the geometric relationship between strain and displacement combined with reference-free calibration that identifies an optimal calibration factor for the strain and acceleration measurements. The obtained strain-based displacement is fused with acceleration data by using an AKF to recursively update the noise covariance of strain-based displacement. The proposed method was validated numerically and experimentally on simple beam structures.

The main findings of this study can be summarized as follows:

- A reference-free displacement estimation method using strain and co-located acceleration measurements is developed.
- Numerical simulations were conducted on a simple beam structure under four different cases of RMS noise for strain measurement (i.e., 5%, 10%, 15%, and 20%). The resulting errors were only 4.64, 6.59, 7.42, and 8.06%, respectively, demonstrating the robustness of the proposed method to strain noise.
- The proposed method provides stable responses, regardless of the initial value of strain noise covariance.
- A field applicability test was conducted on a concrete bridge with a truck travelling across it at two different speeds (i.e., 5 km/h and 15 km/h); the proposed method estimated the peak deflection of the bridge with errors of 6.1% and 1.73% for the two speeds, respectively, demonstrating good performance for full-scale bridge displacement measurement.

In conclusion, the ability to perform displacement estimation using only a single strain and acceleration measurement is the most significant advantage of the proposed method compared to the existing techniques. Additionally, the proposed method can be used in cases where conventional displacement sensors are difficult to install. Future study is underway to implement the proposed method on a wireless sensor for long-term autonomous structural assessment.

## Figures and Tables

**Table 1:** Implementation scheme for the AKF.

Initialization at time step *t*_0_ = 0:
${\hat{x}}_{0}=x_{0}$, $P_{0|0}=P_{0}$
$Q_{0}=[Q]q$, $R_{0}=[R]r_{0};r_{0}>0$
At time $t_{k}$, for *k* = 1, 2, 3…, *N_t_*:
Prediction stage for states: Evolution of states and prediction of error covariance. (26a) ${\hat{x}}_{k}^{-}=A{\hat{x}}_{k-1}+Ba_{m(k)}$ (26b) $P_{k}^{-}=AP_{k-1}A^{T}+Q$
Correction step for estimated states:
Residual calculation and measurement noise covariance at time $t_{k}$. (23) $$s_{k}=\{d_{s(k)}-C{\hat{x}}_{k}\}$$ (25) $$R_{k}=\beta R_{k-1}+(1-\beta )(s_{k}s_{k}^{T}+CP_{k}^{-}C^{T})$$ Calculation of Kalman gain. (27a) $$K_{k}=P_{k}^{-}C^{T}{\left(CP_{k}^{-}C^{T}+R_{k}\right)}^{-1}$$ Correction of predicted state using updated observation. (27b) $${\hat{x}}_{k}={\hat{x}}_{k}^{-}+K_{k}(d_{s}{}_{\left(k\right)}-C{\hat{x}}_{k}^{-})$$ (27c) $$P_{k}=(1-K_{k}C)P_{k}^{-}$$

**Table 2:** Material properties and dimensions of the beam model.

Properties	Values
Length	50 m
Depth	2 m
Width	5 m
Mass Density	7850 kg/m^3^
Elastic Modulus	200 Gpa

**Table 3:** Error comparisons for measured displacement in different noise cases.

Strain Noise Percentage	Case 1: 5%	Case 2: 10%	Case 3: 15%	Case 4: 20%
Error	0.0464	0.0659	0.0742	0.0806

**Table 4:** Comparison of errors.

Loading Case	Reference	Proposed Method	Error
5 km/h	3.568 mm	3.786 mm	0.061
15 km/h	3.638 mm	3.701 mm	0.017
